# The impact of burn injury on the central nervous system

**DOI:** 10.1093/burnst/tkad037

**Published:** 2024-02-01

**Authors:** Amira Allahham, Grant Rowe, Andrew Stevenson, Mark W Fear, Ann-Maree Vallence, Fiona M Wood

**Affiliations:** Burn injury research unit, School of Biomedical Sciences, University of Western Australia, 35 Stirling Highway, Crawley, Perth, WA 6009, Australia; Fiona Wood Foundation, 11 Robin Warren Dr, Murdoch WA 6150, Australia; Centre for Molecular Medicine and Innovative Therapeutics, Murdoch University, 90 South Street, Murdoch, Perth 6150, Australia; Burn injury research unit, School of Biomedical Sciences, University of Western Australia, 35 Stirling Highway, Crawley, Perth, WA 6009, Australia; Fiona Wood Foundation, 11 Robin Warren Dr, Murdoch WA 6150, Australia; Burn injury research unit, School of Biomedical Sciences, University of Western Australia, 35 Stirling Highway, Crawley, Perth, WA 6009, Australia; Fiona Wood Foundation, 11 Robin Warren Dr, Murdoch WA 6150, Australia; Centre for Molecular Medicine and Innovative Therapeutics, Murdoch University, 90 South Street, Murdoch, Perth 6150, Australia; Centre for Healthy Ageing, Health Futures Institute, Murdoch University, 90 South Street, Murdoch Perth 6150, Australia; Burn Service of Western Australia, Fiona Stanley Hospital, MNH (B), Level 4, 102-118 Murdoch Drive, Murdoch, Perth, WA 6150, Australia; Burn injury research unit, School of Biomedical Sciences, University of Western Australia, 35 Stirling Highway, Crawley, Perth, WA 6009, Australia; Fiona Wood Foundation, 11 Robin Warren Dr, Murdoch WA 6150, Australia; School of Psychology, College of Health and Education, Murdoch University, 90 South Street, Murdoch, Perth 6150, Australia

**Keywords:** Burn, Injury, Central nervous system, Brain, Spinal cord, Inflammation

## Abstract

Burn injuries can be devastating, with life-long impacts including an increased risk of hospitalization for a wide range of secondary morbidities. One area that remains not fully understood is the impact of burn trauma on the central nervous system (CNS). This review will outline the current findings on the physiological impact that burns have on the CNS and how this may contribute to the development of neural comorbidities including mental health conditions. This review highlights the damaging effects caused by burn injuries on the CNS, characterized by changes to metabolism, molecular damage to cells and their organelles, and disturbance to sensory, motor and cognitive functions in the CNS. This damage is likely initiated by the inflammatory response that accompanies burn injury, and it is often long-lasting. Treatments used to relieve the symptoms of damage to the CNS due to burn injury often target inflammatory pathways. However, there are non-invasive treatments for burn patients that target the functional and cognitive damage caused by the burn, including transcranial magnetic stimulation and virtual reality. Future research should focus on understanding the mechanisms that underpin the impact of a burn injury on the CNS, burn severity thresholds required to inflict damage to the CNS, and acute and long-term therapies to ameliorate deleterious CNS changes after a burn.

Highlights

- Burn injuries can lead to CNS-related sequala, including neural apoptosis, hyperalgesia, and changes in metabolism and cognitive function.
- The main mechanism that underlies CNS changes after burn is thought to be inflammation but may also be linked to metabolic disruption or the acute dysfunction of the blood–brain barrier.
- Recently both pharmaceutical and non-invasive new therapeutic options have been developed to limit the impact of burn on the CNS and improve patient outcomes.

## Background

Burn injuries affect more than just the skin, causing changes to multiple body systems. Burn injuries are caused by an array of mechanisms including scald, contact burns, friction burns, electrical burns and chemical burns, and range in severity and intensity depending on the layers of skin damaged and the extent of the burn on the body’s surface area. The sequelae of burn injuries are numerous, including burn patients having an increased risk of hospital admission for a range of morbidities such as cancer [1], cardiovascular disease [2], respiratory infection [3] and other diseases [4,5], as well as longer admissions to hospital for these morbidities compared to uninjured patients.

One impact of burn injuries that is often neglected when discussing the effects of burn injuries is the effect on the central nervous system (CNS). Burn-induced effects on the brain and spinal cord are not yet fully understood, and little is known about the extent of these changes and their outcomes. Nevertheless, the findings to date suggest that burn injury can lead to a physiological impact on the CNS.

Systematic inflammation is the initial response to burn injury, which is elicited by the proinflammatory markers released by resident immune cells such as macrophages and T-cells that help in repairing the tissue, preventing infection and clearing necrotic tissue from the wound site [6]. These inflammatory markers can infiltrate different organs and cells and hence trigger stress and immune responses that signal the release of molecules related to immune responses.

The CNS is surrounded by the blood–brain barrier (BBB) and the blood–spinal cord barrier (BSCB) which protects it from many of the systemic responses occurring in the body. After burn injury, and in other trauma, the inflammatory response is both large and temporally extensive, resulting in the BBB and BSCB being compromised, and inflammatory markers may infiltrate the CNS [7]. This inflammatory response continues to affect the body in the long term, where it has been shown to affect metabolism, organ function, quality of life and bodily systems, including the immune and nervous system, for years after the burn [8,9]. One possible impact of this inflammatory response coupled with a compromised BBB is on mental health. In the past, studies considered the mental health complications following a burn to be a ‘normal’ product of the injury and a factor that is exclusively psychological or related to the psychosocial impact of the burn, rather than physiological [10]. However, as our understanding of mental health disorders and their relationship to inflammation has changed, the possibility that there is a direct physiological link between burn trauma and mental health is increasing. In this review, we investigate the evidence of a physiological impact of burn injury on the CNS, and how this may be important in long-term health outcomes.

**Figure 1:**
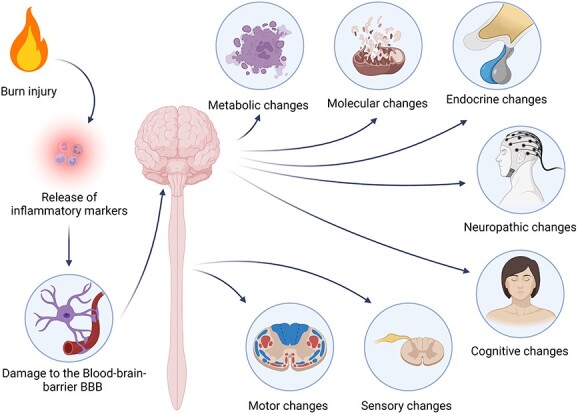
Different areas affected by burn injuries in the central nervous system. Figure was created with BioRender.com

The last review on the impact of burn injuries on the CNS was by Flierl *et al.* [7]. Since then, our understanding of the impact of burn injuries and the mechanisms by which they affect different bodily systems has increased. This review will discuss the changes in the CNS after burn injuries, focusing on both the spinal cord and the brain, the cognitive and functional changes that occur as a result of the changes to the CNS as shown in Figure 1, and a review of the current available treatments and interventions for burn symptoms related to the CNS. Details of the studies mentioned in this review are presented in supplementary Table 1 (see online supplementary material).

## Review

### Impact of burn injury on the BBB

Brain oedema due to severe burns was identified early on as a leading cause of mortality in burns [11,12]. In early case reports investigating the causes of death of burn victims and how their brains were affected after burns, ‘brain swelling’ was identified as the cause of death for paediatric patients [12]. Although the cause of the ‘swelling’ was not identified in the early studies, some morphological changes to the brain were described, including swollen cerebral hemispheres, herniation of the cerebellar tonsils around the medulla, partial demyelination of the cerebrum, flattening of cerebral convolutions, virtual absence of free cerebrospinal fluid and degenerative changes in the cerebellar cortex [11,12]. In an animal model, severe burns [>25% total body surface area (TBSA)] were associated with cellular morphological changes, with cell bodies, nuclei and nucleoli enlargement, uneven contours and the cytoplasm vacuolated [13]. In the intercellular space, it was noted that the matrix became clarified and the cristae were reduced [13].

**Figure 2:**
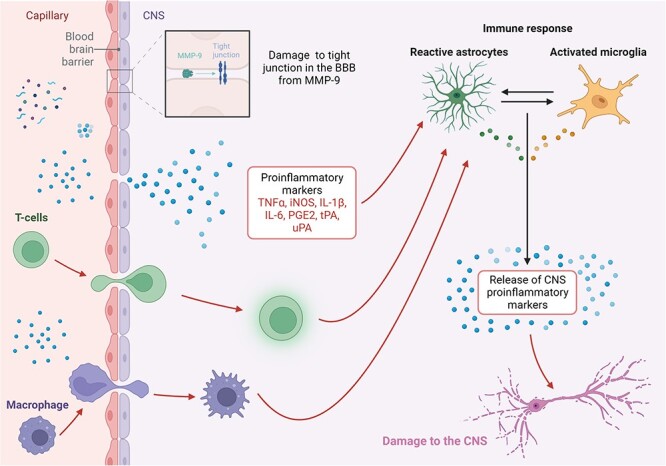
Mechanisms of proinflammatory marker infiltration to the CNS after burn injury and how they can lead to damage in the CNS. *CNS* central nervous system, *iNOS* inducible nitric oxide synthase, *PGE2* prostaglandin E_2_, *tPA* tissue plasminogen activator, *uPA* Urokinase plasminogen activator, *IL-6* interleukin 6. Figure was created with BioRender.com

Burn injuries cause an increase in the level of inflammatory markers in the body [7]. This inflammatory response can increase the levels of proteins and other inflammatory factors released in response to the burn that can cause damage to the extracellular matrix of the BBB causing it to increase its permeability [14]. The increased permeability of the BBB can then lead to increased water content in the brain and thus brain oedema [15]. Matrix metalloproteinases (MMPs) are synthesized in large amounts as a result of the inflammation from burns, and activated MMP-9 in particular can damage the tight junctions in the extracellular matrix tissues, therefore damaging its basal lamina and increasing the permeability of several organs including lungs, skeletal muscles [16] and the brain [17–20]. MMP-9 mRNA is significantly elevated as early as 3 h post a 50–70% TBSA burn, and remains high until 7 h, then returns to normal 24 h post-burn [17]. However, MMP-9 protein levels are elevated at 7 h and remain high until 72 h post-burn [20]. The brain water content measured in these studies was shown to be significantly elevated at 7 h and it remained elevated until 72 h post-burn, indicating that elevated levels of MMP-9 are likely associated with the increased permeability of the BBB and may be contributing to the brain oedema observed after burns [17,20]. A study by Hu *et al.* [21] identified the earliest time point that the BBB begins to open following a 50% TBSA burn was at 2 h, while intracellular oedema and significant damage to tight junctions of the endothelial cells was observed at 3 h post-burn [21]. In another study that also looked at 50% TBSA burns, histological changes in the endothelial cells in the BBB were observed at 6 h post-burn [22]. In a review of the dynamic relationship between MMP-9 and its inhibitor after burn injuries, MMP-9 was consistently found to be elevated acutely at the burn site, while its inhibitor, tissue inhibitor of metalloproteinase-1 (TIMP-1), had a delayed response, with both MMP-9 and TIMP-1 remaining high for months, as shown by gene expression, protein and activity analysis [16]. These increases in MMP-9 and TIMP-1 become systemic and were detected in the serum up to 14 days after the injury where they likely contribute to BBB breakdown [16]. Several other alterations of proteins and enzymes that have been associated with BBB breakdown after burn injuries include increases in levels of tissue plasminogen activator, urokinase plasminogen activator, interleukin 6 (IL-6), IL-1β and tumour necrosis factor α (TNF-α), and decreases in levels of tight junction proteins, including claudin-5, occludin, and ZO-1 [18,19,23]. A summary of the mechanisms by which inflammatory markers infiltrate the CNS after burn injuries is shown in Figure 2, where proinflammatory markers interact with astrocytes and microglia in the CNS causing them to activate, release more proinflammatory markers in the CNS and contribute to damaging the CNS, as will be discussed.

### Inflammatory markers in the CNS after burns

Once the BBB and BSCB are compromised, a number of proinflammatory markers can infiltrate the CNS and cause further changes. In a rat model, severe scald burns covering 60–70% of the TBSA showed acute increases in serum- and brain-inflammatory markers [24]. For example, a 5–50-fold increase in serum inflammatory markers including TNF-α, IL-1β and intracellular cell adhesion molecules protein levels were detected 7 h post-burn, while a 3–15-fold increase in the mRNA of those markers was found in the brain at 3 h post-burn [24]. These responses likely indicate the commencement of pathophysiological changes in the CNS following a burn injury. Nitric oxide (NO), a messenger molecule that acts as a neurotransmitter in the CNS, was investigated by two studies that showed opposing results, which is likely a product of their different methods of acquiring data. The first study found that there are elevated levels of neuronal NO synthase (nNOS) in the frontal cortex, striatum and hippocampus of animals with 30% TBSA burn injuries at 24 h post-burn, which was calculated using immunohistochemistry [25]. The second study that used a voltametric method that used *in situ* NO measurements in the cortex, found that cortical NO levels in animals with 20% TBSA burns decreased during the first 24 h post-burn [26]. Although these results are conflicting, it is clear that there are changes to the levels of NO in the brain as a result of the burn. Prostaglandin E_2_ (PGE2) is another inflammatory marker that has been found to be affected by burns. PGE2 is an inflammatory mediator in the CNS, and after burn trauma both PGE2 and its precursor cyclooxygenase-2 (COX-2) were elevated in the cerebrospinal fluid and in endothelial cells in the brain 36 h after a severe burn injury on rats (25% TBSA, n = 7) [27]. PGE2 and COX-2 contribute to the inflammatory response in the CNS and have been shown to have neurodegenerative effects on the brain [28]. Therefore, the elevated levels observed in the cerebrospinal fluid may have further pathological effects on the CNS.

The elevation of inflammatory markers in the brain can lead to disruption of cell function. Such changes are often related to immunoreactivity, and therefore may cause cell apoptosis, and in select cases could lead to death [14,29]. One way in which immunoreactivity is affected by burns is through the expression of the intranuclear ubiquitin protein, which is a protein expressed in response to heat shock [30]. Post mortem brains of burn victims have been previously studied for intranuclear ubiquitin immunoreactivity and showed a fatal stress response in the substantia nigra of the midbrain [30]. Ubiquitin is a large molecule that can move from the cytoplasm into the nucleus where it combines with denatured proteins to form macromolecular polymeric aggregates [30]. It is suggested that this occurs due to neurodegeneration from excitotoxicity which is caused by fatal burns. Other large molecules may be similarly activated, hence causing more damage to the substantia nigra, including loss of neurotransmitters, oxidative stress, loss of neurotropic factors and apoptosis [30]. A similar study conducted years later comparing the short- and long-term effects on the brain post-mortem found changes in other proteins likely associated with stress experienced from the fatal burn [29]. When comparing these findings to non-burn related death controls, neuronal single-standard DNA immunopositivity was found following acute burn deaths [29]. Fewer neurons and more glial cells in the parietal cerebral cortex were found in victims who died up to 18 days after their burn injury, while basic fibroblast growth factor and glial fibrillary acidic protein were upregulated [29]. Collectively, these findings indicate that extreme burn injuries that are often fatal can lead to apoptosis of neurons which may be caused by the activation of immune cells, while the increase in glial cells and basic fibroblast growth factor and glial fibrillary acidic protein was suggested to reflect the self-protective responses of the brain in prolonged deaths [29]. Another protein in the brain that was shown to be affected by burn injury is cerebral gelsolin, which plays a role in cell adhesion and apoptosis in the brain and has been shown to be increased in expression after burn injury [31]. Gelsolin plays an active role in the activation of immune and glial cells in the brain, thereby playing a role in inflammation-induced neural apoptosis following burn injury [31]. One way in which the changes in immunoreactivity and apoptosis manifest is through brain oedema which often accompanies severe burns [14,32]. Increased water content in the brain following burns, a characteristic of brain oedema, can be explained by the increased expression of certain membrane proteins in the brain, including MMPs, aquaporins and claudin 5, which can damage the BBB and increase its permeability, leading to apoptosis, as will be discussed later in this review [14].

### Inflammatory changes affecting sensory and motor pathways

Burn-induced inflammatory changes in the spinal cord are often linked to the motor and sensory pathways in the brain and can be observed through symptoms in the periphery, such as allodynia and changes in motor function. In a study that investigated dendritic spine density in ipsilateral spinal motor neurons in burn-injured animals, spinal cord changes can present as increases in dendritic spines in the motor neuron soma, which is a morphological characteristic associated with increases in neuronal excitability [33]. Changes at the histone level in the dorsal horn spinal neurons have also been observed shortly after burn injuries and may be involved in the development of central sensitization which can lead to changes in neuronal excitability [34]. As a consequence, tactile allodynia may develop, i.e. survivors experiencing painful symptoms when exposed to innocuous stimuli [35]. Burn injury-induced central excitability changes due to reduced CNS neuronal activation thresholds can occur due serum inflammatory markers, while other proteins and molecules also contribute to this excitability change, including potassium ions [36], extracellular signal-regulated kinase and metenkephalin in the spinal horns [37,38], and spinal p38 mitogen-activated protein kinase (MAPK) in microglial cells [35]. p38 MAPK can further induce the synthesis of TNF in the spinal cord, to which has been attributed the hypersensitivity and burn pain in the early stages of the burn, while the maintenance of chronic pain after burn injury has been attributed to other molecules such as c-Jun N terminal kinase MAPK-dependent chemokine [39].

### Changes in the hypothalamic–pituitary–adrenal axis

Burn injuries can also cause CNS-related changes to the endocrine system, particularly in the hypothalamus and pituitary gland. While there is limited research that has investigated the burn-induced effects of the endocrine system, Moiseev *et al*. [40] reported changes in the neurosecretory functions of the hypothalamus, while Stoner and Elson [41] showed a decrease in the hypothalamic noradrenaline concentration. Several feedback loops in the hypothalamic–pituitary–adrenal (HPA) axis were found to be altered after burn injuries, including the negative feedback loop between serum cortisol, adrenocorticotropic hormone and dehydroepiandrosterone sulfate with vasopressin [42]. Other researchers have found temporary early disturbance in cortisol secretion in a small number of severe burn patients (n = 7), which can lead to adrenal insufficiency after severe burns [43]. Research conducted on male adult mice examined the combined effect of alcohol and burn injury on the hypothalamus. Hypothalamic concentrations of inflammatory cytokines such as TNF-α, IL-6 and IL-1β, and luteinizing hormone releasing hormone were significantly increased in mice that received burn injuries, mice that received alcohol and mice that received both, while serum testosterone was significantly decreased in all three groups [44]. However, there was no additive effect on the concentrations of inflammatory markers in the hypothalamus in the group where mice received both burn and alcohol interventions [44]. Together, this suggests an impact of burn trauma on the HPA axis, providing a mechanism by which burn trauma not only impacts on the CNS directly, but the CNS then acts to mediate more widespread systemic effects across body systems through the HPA axis.

### Metabolic changes in the CNS

Several metabolic processes involving the mitochondria and the synthesis and uptake of glucose are affected by burn injury. Elevated levels of stress hormones such as catecholamines, cortisol and glucagon following burn injuries are suggested to cause a decrease in mitochondrial glutathione levels [45]. Mitochondrial glutathione is an intracellular antioxidant that can produce reactive oxygen species in the cerebral cortex [45]. Glucose metabolism is also altered after burn injury. Research using a mouse model detected burn-induced changes in mice who received a lipopolysaccharide injection 7 days after the burn injury [46]. Burn injury predisposed mice to lipopolysaccharide-induced changes in glucose metabolism, where the positron emitting tracer, [^18^F]2-fluoro-2-deoxy-glucose, which can help measure metabolic activity, was shown to be decreased in the brain both 7 days after the burn in mice that only received the burn and 3 h after lipopolysaccharide injection in mice that received both the burn and the injection [46]. Glucose utilization in the brain using [^18^F]2-fluoro-2-deoxy-glucose is also decreased at 6 and 24 h after the burn but levels return to normal after 3 weeks [47]. It is also reported that there is a decrease in oxygen consumption and hexokinase activity, and an increase in glucose-6-phosphatase activity *in vivo* [47]. The expression of glucose transporter GLUT1 is also altered after burn injury, where it was found to be elevated in the brain from 4–72 h following burns, both at the mRNA level and at the protein level, indicating disturbances to glucose uptake and glycolysis [48]. Alterations in feeding, energy expenditure and brain amine neurotransmitters are also reported in rats that had 30% TBSA burn injuries who experienced anorexia for 4 days where they did not consume food after the burn injury, followed by hyperphagia beginning on postburn day 10, whilst dopamine metabolism was increased in the corpus striatum, nucleus accumbens and amygdala, and norepinephrine levels were elevated in the hypothalamus and nucleus accumbens, indicating hypermetabolism in the brain [49]. It was theorized that the elevated dopamine metabolism is associated with the anorexia observed, while the hyperphagia–hypermetabolism may be mediated by norepinephrine [49].

Other metabolic changes following burns include the release of lipid agonists that are oxidized via oxidative enzymes and can in turn activate transient receptor potential (TRP) channels, TRP vanilloid 1 (TRPV1) and TRP ankyrin 1 (TRPA1), that are involved in the central mechanism of allodynia or persistent pain after burn injuries [50]. Therefore, the release of the oxidative enzymes that influence TRPV1 and TRPA1 contributes to hypersensitivity and mechanical and thermal allodynia [50]. Disruptions in the signalling pathways at the molecular level in the brain after burn injuries can also cause pathophysiological and metabolic changes. Burn injuries can induce insulin resistance and encephalopathy in the early stages of burns through the activation of MAPKs [51]. The stress response from the burn injury can also disturb glucose transport and protein synthesis, which in turn can lead to insulin signal transmission and insulin resistance [51].

### Functional changes affecting the periphery

Several areas of the brain are affected by the burn, and interestingly, functional changes to certain regions of the brain reflect the brain’s plasticity in accommodating the changes in bodily functions. For example, functional near-infrared spectroscopy brain activation differences between survivors and healthy controls were detected when walking [52]. The prefrontal cortex of burn survivors had heightened activity compared to healthy controls, indicating that more attention (accommodation) was required to complete walking tasks [52]. Synaptic and muscle mass changes were also reported in the spinal cord which are associated with the activation of microglia in the CNS [53]. Central and peripheral neural pathways have a dynamic relationship, as demonstrated in full-thickness burn injuries that were shown to cause apoptosis of spinal cord ventral horn motor neurons, which in turn lead to muscle apoptosis and denervation atrophy in the area of the burn; therefore damage from the burn causes apoptosis in the central pathways which in turn causes muscle atrophy in the periphery [54]. These central changes in the motor pathway can lead to motor dysfunction that continues to affect burn patients long after their injury. Burn patients continue to suffer from limitations to movement and motor functions, with symptoms including joint pain and stiffness, problems in walking or running, fatigue, and weak arms and hands, and these symptoms continue to be reported at an average of 17 years after burn injury [55].

#### Pain-inducing changes

Central sensory pathways are impacted by burn injuries causing changes in neuron firing and hence pain. Hypersensitivity to pain after burn injury is a central mechanism that is caused by the denervation of the injured region [56]. Neuropathic pain, which is common after burn, can present itself in different symptoms including mononeuritis multiplex, mononeuropathy, radiculopathy and generalized axonal polyneuropathy [57]. There is evidence to suggest that neuropathic pain is associated with central mechanisms in the dorsal roots of the spinal roots that cause increased nociception and hence pain. Diagnosed neuropathic pain can be treated with Pak1 inhibitors, which are Rac1 effector kinases that link Rac1 signalling to the cytoskeletal reorganization underlying dendritic spine plasticity [58]. Rac1 maintains allodynia symptoms by affecting the density of abnormal dendritic spines on the dorsal root of the spinal cord [59]. Rac1 inhibitor was found to decrease mechanical allodynia and electrophysiological signs of burn-induced neuropathic pain; however, it only partially restored the normal thresholds of pain, as inflammation also likely plays a part in the increased levels of pain [59].

One of the earlier steps of hyperalgesia is activation of *N*-methyl-D-aspartate (NMDA) receptors and the consequent activation of Ca^2+^-dependent second messenger cascades which in turn affect synaptic plasticity [60]. However, burn-induced secondary hyperalgesia is dependent on Ca^2+^-permeable α-amino-3-hydroxy-5-methyl-4-isoxazolepropionic acid (AMPA), but not NMDA receptors, where blockage of AMPA voltage-gated calcium channels via specific antagonists has shown potential in reducing neuropathic pain due to burn injuries [60]. In contrast, secondary messengers and retrograde neurotransmitters of NMDA receptors which also act as inflammatory markers, such as spinal NO, PGE2 and its precursor COX-2, were shown to not be involved as second messengers downstream of Ca^2+^-permeable AMPA receptors [60]. However, there is evidence that NMDA antagonists help attenuate mechanical allodynia and thermal hyperalgesia [61]; therefore, there are likely multiple mechanisms involved in the incidence of neuropathic pain after burns and as such there are multiple treatment pathways to consider to relive burn patients of the pain.

Neuropathic pain may become chronic due to metabolic mechanisms related to the mitochondria and its regulators such as proliferator-activated receptor-gamma coactivator-1. Proliferator-activated receptor-gamma coactivator-1 can cause increases in mitochondria density of central pathways which may cause prolonged nociception after burn injuries [62]. Neurotropic pain may also present itself as allodynia, which has been shown to be related to glutamatergic receptors in the dorsal horn of the spinal cord such as amino-3-hydroxy-5-methyl-4-isoxazole propionic acid–kainate [63]. Chronic exposure to stress prior to the burn injury may also contribute to allodynia, where brain-derived neurotrophic factor (BDNF) signalling in certain parts of the brain, such as in the prefrontal cortex and the hippocampus, has been shown to alter basal pain thresholds which alleviates allodynia after burns [64]. Other psychological stressors such as sound stress can also alter the thresholds of pain after burn injury and contribute to allodynia through several mechanisms involving the release of cortisone through the HPA axis which alters signal transmission of nociception [65]. When the neurons at the injury site have increased firing due to their damage, the input from these neurons sensitizes both the injury site and the central pathways of the spine as there is a large input from the injured site, and due to the sensitization of those spinal pathways, hypersensitization occurs in the surrounding tissue of the burn site [66].

#### The aetiology of burn injury and its importance in CNS sequelae

The functional impact of the specific aetiology of burn injury can cause distinct sequela. Electrical injuries are a type of burn whereby a high voltage electrical current is conducted throughout the body and the areas where the conduction began are often severely burnt. Electrical burns have more complicated neurological sequela compared to burns by other mechanisms. One symptom of electrical burn is clonus, which is a late onset neurological sequela of electrical burn injury that is categorized as a series of rhythmic, monophasic contractions and relaxations of a group of muscles [67]. Clonus is likely caused by the conduction of current through body systems in the neurological pathways of the injury site, but often extends beyond these pathways leading to cerebral, spinal or peripheral neural complications [67]. Chemical injuries are caused by contact with highly acidic or highly alkali substances, and many such injuries involve eye burns [68]. Patients often experience ocular pain after chemical eye burn injuries and are often diagnosed with dry eye symptoms. However, traditional medications for dry eye do not work after burn injury due to the source of the pain being from neuronal injury to eye nerves [69]. In a study that looked at the effect on CNS pathways after chemical eye injury due to an alkali burn, extracellular signal-regulated kinase, a marker for neuronal activation in chronic pain processing, was found to be elevated in different regions of the CNS, including the insular cortex, anterior cingulated cortex and rostroventral medulla [69]. Alkali burns activate central pathways of pain post-burn, which cause the spontaneous pain experienced by the burn, and these central mechanisms are likely involved with the maintenance of pain and its development into chronic pain [69].

### Cognitive changes

With the extensive impact that the burn has on the nervous system, there are inevitable changes to cognition which can arise either from the physiological changes caused by the injury to the CNS or from the psychological impact from the incident of the injury and its social impact on patients’ lives. The depletion of NO levels after the burn injury may be associated with some cognitive changes observed in the acute stages of the burn [70]. NO is an important agent in memory formation, and in a study that tested the memory of mice after burn injury and their NO levels in the first few days after the burn, NO levels were found to be depleted in the first hours of the injury and then stabilized at a third less than normal levels; at the same time, memory was tested via recognition of familiar objects and found to be worse than that of the controls, indicating that NO levels are likely involved in the changes in memory [70]. The decrease in NO levels may also lead to other cognitive and behavioural changes as NO is involved in learning behaviour. However, the mechanism of its depletion is not fully understood [70]. Previous reports demonstrated that nNOS levels are increased in the brain following burns, therefore the mechanism of its metabolism and the relationship between NO in the CNS and in the periphery at the injury site requires further research [7,25,70]. Another study that looked at long-term changes (3 months) in behaviour after burn injury in mice found that mice with burn injuries have increased locomotion, which is indicative of a high arousal state, and their spatial memory was improved in contextual fear-conditioning tasks compared to sham mice, while the auditory fear memory was impaired in burn mice compared to sham [71]. Furthermore, bad mood in mice with burn injuries was demonstrated in a social interaction task where mice with burns had a deficit in sociality and a preference for social novelty as compared to sham mice, a behaviour similar to that of mice with PTSD, while sham mice preferred social interaction [71]. These changes in behaviour were also accompanied by the elevation of inflammatory biomarkers including activation of astrocytes and microglia in the hippocampus, and interestingly, the elevation of BDNF levels [71]. BDNF plays a role in synaptic plasticity in the hippocampus that helps in memory extinction in fear situations, but in models of trauma neurodegeneration, BDNF is often elevated in the brain except in models of stroke, orthopaedic surgery and traumatic brain injury that all have a prolonged activation of microglia. Similarly, a prolonged activation of microglia was also found 3 months after burn injury. This elevation in BDNF was suggested to serve as a compensatory mechanism for brain injury as a result of the persistent activation of microglia, and therefore may potentially lead to maladaptive plasticity [71].

The CNS induces the release of certain neurotransmitters following burn injuries which help promote healing, re-epithelialization and immune processes at the burn site [72]. Neurotransmitters such as serotonin, epinephrine, glutamate and dopamine influence immune cells and inflammatory markers such as cytokines at the wound site as they mediate the interaction between the immune networks and the CNS, therefore promoting regeneration after burn injuries [72]. In the brain, a study on regional concentrations of neurotransmitters in guinea pigs with 30% TBSA burns showed that there was elevation of serotonergic neurotransmission, reduction of dopamine metabolism and elevation of norepinephrine concentrations in brain areas thought to control feeding [73]. Another study on mice with 5% full-thickness burns, showed that mice had increased concentrations and transcription of monoamine oxidase A (MAOA) a week after their burn injuries, which is a neurotransmitter associated with CNS abnormalities related to depression [74]. Interestingly, this study also showed that mice had depression-like behaviour, which was decreased with treatment with a MAOA inhibitor, indicating that the role of the MAOA transmitter is also related to cognitive functional changes that occur after burn injuries [74].

Cognitive impairment after burn injuries has also been demonstrated in patients. In a study that used data from the Uniform Data System for Medical Rehabilitation, burn patients undergoing rehabilitation scored significantly lower on the cognitive functional impairment scale of the functional independence measurement instrument compared to other populations undergoing rehabilitation, including patients with spinal cord injuries, amputations, multiple fractures and hip replacement [75]. Interestingly, the subscale driving this difference in cognition was memory, which is suggested to be due to the sensitivity of the hippocampus (which is involved in the formation of memories) to the stress induced after burn injuries [75]. Physiological stress, like that induced by burn injuries, can impact the morphology of the hippocampus by affecting its dendrites, and inhibits its neurogenesis which is responsible for creating new memories [76]. In the study by Purohit *et al.* [75], burn patients also scored lower on other subscales in the cognitive domain, including problem solving, comprehension, expression and social interaction, indicating that burn injury likely has a more severe impact on cognition compared to the other injuries due to the extensive inflammatory response involved [75]. There are many factors to consider that may be affecting these differences, including the medications taken while in rehabilitation, diet, surgical procedures, fluid intake and electrolyte disturbance. Therefore it is hard to determine whether the changes in cognitive ability are due to the physiological impact that the burn has had on the body or from factors associated with the recovery period [75]. Supplementary Table 1 shows a summary of all publications discussed, outlining the models used and the key findings regarding the impact of burn injuries on the CNS.

Another cognitive complication of burn injuries that often arises in the acute stages of burn care, especially in patients with severe burns in the intensive-care unit, is delirium [77]. Delirium is a neuropsychiatric syndrome that often occurs in conjunction with surgeries, drug use and withdrawal, or trauma, and it is characterized by reduced cognitive consciousness and altered arousal, from reduced responsiveness at a near-coma level to hypervigilance and severe agitation [77,78]. Due to the severity of their injuries, burn patients often receive large dosages of analgesic and sedative medications during their surgeries and recovery, which are associated with an increased risk of delirium [79]. The combination of medications such as benzodiazepines and pain, increases the risk of developing delirium in the intensive-care unit, however, other medications such as opiates and methadone were suggested to reduce the risk of developing delirium as they reduce the pain for burn patients [79]. Delirium is caused by alterations in several processes in the CNS, including changes in brain connectivity, and neuroinflammatory and vascular changes, all of which occur as a result of brain vulnerability due to multiple factors including a compromised BBB [78]. Although most studies on delirium focus on its acute stage that often occurs during patient care, delirium may have long-term consequences involving long-term cognitive impairment which needs to be further explored [80].

### Treatment of CNS changes induced by burn injury

**Figure 3:**
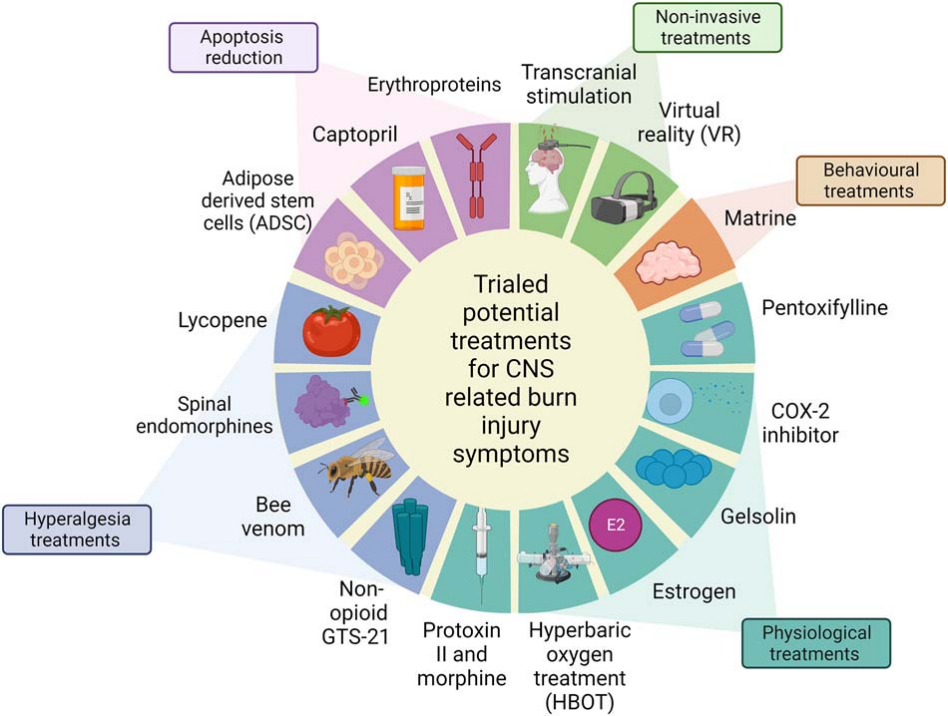
CNS-related treatments of burn injury symptoms that include invasive and non-invasive treatments and their categories. *CNS* central nervous system, *COX-2* cyclooxygenase-2. Figure was created with BioRender.com

Several treatments have been investigated which promise to alleviate some of the CNS-related symptoms that accompany burn injuries, including both invasive and non-invasive treatments. Treatments discussed in this section are highlighted in Figure 3.

#### Invasive treatments

The treatment of the symptoms and complications of burn injury that arise from changes in the CNS are often done in the recovery stage. Several methods have been trialled to treat neuropathic pain after burn injuries, including hyperbaric oxygen treatment (HBOT) [81,82], bee venom [83], adipose-derived stem cells (ASCs) [84,85], lycopene [86], matrine [87], spinal endomorphins [88] and non-opioid GTS-21 [89]. HBOT is a method used to promote wound healing whereby patients are treated with 100% oxygen to reduce inflammation. A reduction in neuropathic pain was observed following HBOT compared to a burn control group, where pain levels returned to normal levels for at least 3 weeks after the treatment [81] Upregulation of melatonin and opioid receptors in several regions in the brain and downregulation of BDNF, substance P and calcitonin gene-related peptide were also observed in the HBOT group [81]. Early treatment with HBOT was also found to reduce inflammatory markers including TNF-α, IL- 1β, galectin-3, toll-like receptor-4, CD68 and CD45 in the dorsal horn of the spinal cord [82]. Bee venom is another novel treatment that has shown positive results on neuropathic pain. Bee venom has natural toxins with anti-inflammatory effects and it is often used in alternative medicine to treat a range of diseases [83]. Bee venom can alleviate neuropathic pain after burn injury when administered repeatedly in 0.02 or 0.1 mg/kg doses in mice (n = 7). For example, bee venom significantly reduced the area of tissue damaged over time and reduced the inflammatory marker substance P in the dorsal root ganglion of the spinal cord, indicating that the treatment improved both central and peripheral pathways [83]. The cutaneous transplantation of autologous ASCs also showed a reduction in inflammatory markers in the spinal cord after burn injury including COX-2 and NOS, and mechanical thresholds were also significantly reduced after the treatment, indicating reduction in pain levels [84]. Transplantation of ASCs can significantly attenuate apoptotic death in the ventral horn of the spinal cord, however, the mechanisms by which this happens and how ASCs can reduce burn-induced inflammation are not yet understood [85].

Lycopene is a compound of the carotenoid family that is found in some fruits and vegetables such as tomatoes, and has been shown to be involved in upregulating the expression and activity of the sirtuin 1 gene and the mammalian target of rapamycin pathway that are involved in reducing reactive oxygen species and regulating cell proliferation and immune cell differentiation [86]. Lycopene can reduce neuropathic pain after burn injuries by significantly reducing mechanical pain thresholds, reducing glial activation in the dorsal root ganglion of the spinal cord, and altering sirtuin 1 and mammalian target of rapamycin pathways, as demonstrated in a mouse model (n = 8) [86]. Intrathecal administration of spinal endomorphins was also shown to attenuate burn pain as it inhibits the phosphorylation and hence activation of the p38 MAPK pathway and has potent analgesic effects on spinal pain pathways mediated via the mu-opioid receptor-dependant p38 MAPK [88]. Nicotinic α7acetylcholine receptors are expressed in the microglia during muscle wasting and are important in pain behaviour [89]. GTS-21 is a highly selective agonist of nicotinic α7acetylcholine receptors, and its administration after burn injuries has shown anti-inflammatory effects, attenuating the exaggerated nociception at the injury site and after 14 days of administration accelerating recovery from pain [89].

As it is now understood that severe burn injuries have a long-lasting effect on the CNS, many treatments focus on reducing the inflammatory effects of the burn on the CNS. The early stages of the burn (~7 h) is when the highest levels of inflammatory markers are released, hence, it may be an effective window for potential treatments [24]. After early administration (15 min) of pentoxifylline, a phosphodiesterase inhibitor with anti-inflammatory effects, after burn injury, the activation of proinflammatory cytokines such as TNF-α, IL-1β and IL-6 was found to be suppressed, which resulted in the suppression of the activation of microglia and astrocytes in the brain compared to burns without the pentoxifylline treatment [90]. Gelsolin is another substance that was shown to reduce the effects of inflammation on the CNS after burns. Gelsolin is an actin-binding protein that has also been shown to be involved in the systemic immune response, and has protective effects on the CNS following burn injury, including amelioration of pathological lesions and suppression of microglial activation [91]. Gelsolin was also found to downregulate the release of early proinflammatory cytokines such as IL-6 and IL-1β, and late proinflammatory cytokines such as high mobility group box-1 protein, which was suggested to decrease the effects of cell loss and cortical damage in the long-term [91]. Oestrogen was also found to reduce cytokine release in the CNS after burns by reducing their signalling via interacting with transcription factors in a receptor-dependent manner, and hence downregulating their expression [92]. Furthermore, oestrogen was shown to modulate the effects of MAPK and extracellular signal-regulated kinase signalling, which in turn can decrease cytokine signalling and subsequent cytokine production [92]. Treatment with erythropoietin, which is involved in the activation of different kinases and intracellular signalling pathways in the CNS, was also found to reduce programmed cell death and microglia activation, and it even reduced the expression and activation of inducible nitric oxide synthase and COX-2 in the ventral spinal cord after burn injuries [93].

Sodium channels are one of the main components of signal transduction between neurons, and in burn-induced exaggerated nociception, the sodium channel Nav1.7 is upregulated in primary sensory neurons in the spinal cord [94]. Protoxin II is a Nav1.7 channel inhibitor, and together with morphine, it was shown to reduce the burn injury-induced upregulation of both phosphorylated serine 10 in histone H3 and phosphorylated extracellular signal-regulated kinase 1/2, which are both markers for spinal nociceptive processing [94]. PGE2 and its precursor COX-2 are another target of treatment of burn-induced inflammation in the CNS, where treatment with a COX-2 inhibitor has been shown to reduce the central mechanisms of hyperalgesia and is suggested to improve the regulation of the HPA axis hormones in the CNS after burn injuries [27]. Captopril, an angiotensin-converting enzyme inhibitor that is often used to treat heart conditions such as hypertension, has been found to have anti-apoptotic properties where it was shown to decrease apoptotic cells in the cerebellum and the midbrain [95]. In a study mentioned previously on nNOS levels in the brain after burn, treatment with captopril was found to reverse the increase in nNOS activity in the brain. However, this treatment only decreased the level of activity in some brain regions tested, such as the frontal cortex and the midbrain, whilst in the striatum, the levels of nNOS activity were elevated [25], indicating that captopril may have different effects on different brain regions after a burn.

With the wide range of symptoms and molecular and cellular impacts that the burn can cause to the CNS, treatments specific to symptoms with CNS pathways are now in development, including treatments for cognitive and behavioural changes after burns. A study investigated the effects of matrine, a quinolizidine alkaloid with anti-nociceptive, anti-inflammatory, anti-Alzheimer’s, anti-Parkinson’s and anti-epileptic properties, on the depression-like and anxious behaviour of mice after burn injury [87]. The study found that matrine reversed the changes in behaviour after burn injury where it had antidepressant/antianxiety effects that were achieved by targeting c-Jun N terminal kinase-mediated apoptosis and BDNF/vascular endothelial growth factor signalling in mice after burns [87].

#### Non-invasive treatments

Non-invasive treatments provide a promising new avenue for burn pain management and attenuation of CNS-related symptoms including pain and itch [96]. For example, the use of virtual reality (VR) during wound care showed an increase in cerebellar perfusion in burn patients who experienced VR compared to healthy norms, however, the increase was greater in patients who did not experience VR compared to those who did, indicating that VR has the potential to alter patients’ experiences for managing pain during wound care [97]. VR was also shown to modulate brain activity in multiple areas, as demonstrated by various brain imaging techniques including functional magnetic resonance imaging [98]. Augmented reality, which is another form of VR that allows users to interact with virtual elements in real life, has also been shown to decrease pain scores significantly during wound dressing in paediatric burn patients [99].

Transcranial magnetic stimulation (TMS) and transcranial direct current stimulation (tDCS) are neurostimulation treatments that utilize the brain’s plasticity to treat a range of conditions from neuropathic pain to depression [100,101]. TMS is a non-invasive technique that uses brief high-intensity magnetic fields to induce currents and thus depolarize neurons in small regions of cortex, and when delivered in repeated trains of stimulation can induce plasticity for therapeutic purposes to treat neurological and psychiatric conditions [102,103]. TMS has been used to investigate changes in motor functions following burn injuries, where it was shown to be a good method of investigating cortical inhibition following burn injury as it detected abnormalities in conduction pathways in burn patients as well as changes in neuroplasticity in different stages of their treatment [104–107]. Although TMS has not yet been used as a treatment for burn patients, its potential to treat neuropathic pain suggests that it could serve as a potential treatment for CNS-related changes in burn patients, including the neuropathic pain that accompanies the burn, and so future studies should aim to investigate its potential. On the other hand, tDCS is another neurostimulation technique that induces neuroplasticity by delivering weak direct current through surface scalp electrodes, which modulate human brain activity by increasing or decreasing excitability of certain cortical regions [108,109]. One study examining the efficacy of tDCS delivered to M1 to treat post-burn neuropathic pain and itch showed an interaction between pain and itch, with active M1 tDCS disrupting sensory compensatory mechanisms, with no therapeutic effects in burn patients with itch and pain symptoms [96]. It was suggested that targeting other brain regions, such as the prefrontal cortex, where downregulation of affective conditions such as pain and modulation of the emotional experience of pain occurs, may have better therapeutic outcomes for burn patients [96]. Several targets for non-invasive cortical stimulation treatments for burn patients have been suggested to treat specific burn-related symptoms, including the prefrontal region to treat pain and other psychosocial symptoms, the parietal area to treat severe cognitive fatigue and the motor cortex to treat motor dysfunction. Furthermore, tDCS (and TMS) protocols may be effective for mental health complications that are evident following burn injury, such as depression [110].

## Conclusions

Burn injuries have widespread sequela that affect many body systems including the CNS. In this review we have explored the different ways that a burn affects the CNS. There is now significant and accumulating evidence, gleaned from animal and human studies, that burn injury causes a sustained physiological impact on the CNS. The overarching mechanism underpinning the burn-induced changes in the CNS has not been elucidated, although it appears that inflammation and metabolic aberration effects are critical. One thing that remains unknown are the thresholds of burn severity needed to cause CNS changes, and factors such as burn depth, burn extent (TBSA %), burn location and how innervated it is, and other injury- and patient-related factors such as surgeries required and predisposition to neuronal effects, make it difficult to set a standard burn threshold to predict the occurrence of CNS changes after burns. With post-mortem analyses contributing significantly to what is known about CNS changes following burn injury, more research innovation is required to further our understanding about the CNS in burn survivors. For example, research should focus on understanding the triggers impacting burn-induced CNS damage, the long-term neural consequences of burn injuries on the CNS, as well as the impacts of invasive and non-invasive treatments targeting the CNS in burn survivors’ recovery. This may lead to improving long-term health outcomes for burn patients.

## Supplementary Material

Supplementary_table_1_tkad037

## Abbreviations

AMPA: α-Amino-3-hydroxy-5-methyl-4-isoxazolepropionic acid; ASC: Adipose-derived stem cells; BBB: Blood–brain barrier; BDNF: Brain-derived neurotrophic factor; BSCB: Blood–spinal cord barrier; CNS: Central nervous system; COX-2: Cyclooxygenase-2; HBOT: Hyperbaric oxygen treatment; HPA: Hypothalamic–pituitary–adrenal; IL-1β: Interleukin 1-Β; MAPK: mitogen-activated protein kinase; MMPs: Matrix metalloproteinases; NMDA: *N*-Methyl-D-aspartate; nNOS: Neuronal nitric oxide synthase; NOS: Nitric oxide synthase; PGE2: Prostaglandin E_2_; TBSA: Total body surface area; tDCS: Transcranial direct current stimulation; TIMP-1: Tissue inhibitor of metalloproteinase-1; TMS: Transcranial magnetic stimulation; TNF-α: Tumour necrosis factor α; TRPA1: Transient receptor potential ankyrin 1; TRPV1: Transient receptor potential vanilloid 1; VR: Virtual reality.
