# Supplementary material for: The impact of burn injury on the central nervous system
Source: Burns Trauma. 2024 Feb 1;12:tkad037. doi: 10.1093/burnst/tkad037 (PMC10835674; doi:10.1093/burnst/tkad037)
Supplement: Supplementary_table_1_tkad037 [file supplementary_table_1_tkad037.docx]

**Supplementary table 1.** A list of the studies in the review paper elaborating on the experiments done to investigate the effect of burn injuries on the CNS.

|  | **Authors, year, and country** | **Study title** | **Species, n** | **TBSA** | **Mechanism of testing** | **Findings** |
| --- | --- | --- | --- | --- | --- | --- |
| Impact on the Blood-Brain Barrier (BBB) | | | | | | |
|  | Baek (1987), Russia | Ultrastructural changes in the supraoptic nucleus and posterior lobe of the hypophysis following experimentally induced burns | Dogs, 10 | 25% | Light microscopy | Structural changes in cell organelles |
|  | Barone et al. (2000), USA | In vivo visualization of cerebral microcirculation in systemic thermal injury | Rats, 12 | 70% | Vascular leakage of Bovine Albumin and fluorescein isothiocyanate | Increase in cerebral permeability from 20% at 15 mins to 104% at 6 hours |
|  | Berger et al. (2007), USA | Peripheral thermal injury causes early blood–brain barrier dysfunction and matrix metalloproteinase expression in rat | Rats, 35 | 70% | Evans blue analysis for BBB leakage, measurement of brain water content, and MMP mRNA analysis using PCR | Water content and BBB permeability significantly increased at 7 hours, and expressions of MMP increased at 3 hours and remained high at hour 7 |
|  | Emery and Campbell Reid (1962), UK | Cerebral oedema and apastic hemiplegia following minor burns in young children | Humans, 7 | 8-30% | Necropsy | Burn victims had most severe brain herniation through foramen magnum. One case had necrosis of the cerebellar tonsils. |
|  | Hu et al. (2016), China | Dynamic Observation on Opening of the Blood–Brain Barrier in the Primary Stage of Severely Scalded Rabbits, a Multimodal Study | Rabbits, 30 | 50% | 25I-labeled bovine serum albumin to identify earliest time point of BBB opening after severe scald. Dual source CT and magnetic resonance. Light microscopy. | BBB opening at 2 hours post burn. Intracellular oedema and breakdown of tight junction of vascular endotheliocytes at 3 hours post burn. 125I-labeled bovine serum albumin had increased intake at 3 hours. Diffusion coefficient decreased at 4 hours post burn |
|  | Li et al. (2009), China | Stereoscopic study on capillary density of early brain oedema in a dog postburn model | Dogs, 26 | 50% | MRI and histopathology | Cerebral oedema at 12 h, histological changes at 6 h. Swelling of endothelial cells and peri-vascular astrocytes, and vacuolation in neurons at 12 h. Increase in cerebral water content at 6 h. Distributional density of capillaries became thicker at 6 h and 12 h postburn, the shapes were normal. Capillaries became sparser at 18 h. |
|  | Patel et al. (2008), USA | Blood brain barrier (BBB) dysfunction associated with increased expression of tissue and urokinase plasminogen activators following peripheral thermal injury | Rats, 46 | 60-70% | Evans blue, water content in the brain, levels of tPA and uPA mRNA in brain using PCR | Increase in brain water content and presence of Evans blue in brain tissue, temporally associated with increased expression of endogenous tPA and uPA |
|  | Reyes et al. (2009), USA | Role of tumor necrosis factor–α and matrix metalloproteinase–9 in blood-brain barrier disruption after peripheral thermal injury in rats | Rats, 32 | 60-70% | TNF-α neutralizing antibody or doxycycline injection at 8 h, fluorescein isothiocyanate–dextran and water content analysis, Western blot | MMP-9 enzyme activity was significantly increased after thermal injury but decreased in the presence of either TNF-α antibody or doxycycline |
|  | Swann et al. (2007), USA | Peripheral thermal injury causes blood–brain barrier dysfunction and matrix metalloproteinase (MMP) expression in rat | Rats, 72 | 60-70% | Brain water content. Real time PCR, Western blot, and zymography to quantify MMP mRNA, protein, and enzyme activity levels. | Brain water content was significantly increased 7-72 h after burn. Expression of brain MMP-9 mRNA increased at 3 h, remained at 7 h, and returned to control levels by 24 h. MMP9 protein levels and enzyme activity began to increase at 7 h and reached significant levels between 7 and 24 h after burn. While MMP-9 protein levels continued to increase significantly through 72 h, enzyme activity returned to control level. |
|  | Wang et al. (2013), China | Molecular pathology of brain edema after severe burns in forensic autopsy cases with special regard to the importance of reference gene selection | Humans, 55 | NA | Post-mortem mRNA and immunohistochemical expressions of aquaporins (AQPs), claudin5 (CLDN5), and matrix metalloproteinases (MMPs) | Prolonged deaths due to severe burns had increase in brain water content, relative mRNA quantification higher expression levels were detected for all markers |
|  | Warlow and Hinton (1969), UK | Early neurological disturbance following relativly minor burns in children | Humans, 6 | 9-33% | Case report | burns encephalopathy not rare. Transitory neurological disturbances in many children admitted. Mild clinical changes have been ignored because spontaneous recovery usually occurs within a few hours. |
|  | Yang et al. (2020), China | Burns Impair Blood-Brain Barrier and Mesenchymal Stem Cells Can Reverse the Process in Mice | Mice, >50 | ~10% | BBB permeability evaluated using immunohistochemistry imaging, spectrophotometric quantification, and western blot. IL-6, and IL-1b levels in blood and brain were measured by enzyme-linked immunosorbent assay. Transmission electron microscopy (TEM) used to detect transcellular vesicular transport in BBB. | BBB permeability increased to both 10-kDa and 70kDa dextran. IL-6 and IL-1b levels increased in peripheral blood and CNS. Decreased level of tight junction proteins (TJs), including claudin-5, occludin, and ZO-1, which indicated increased BBB permeability due to paracellular pathway. Increased vesicular density suggested increased transcytosis in brain microvascular endothelial cells. Administering UC-MSCs at 1 h after burns effectively reversed these adverse effects and protected the integrity of BBB. |
| Inflammatory markers in the CNS after burns | | | | | | |
|  | Andreasen (1974), USA | Neuropsychiatric complications in burn patients | Humans, NA | NA | Retrospect follow up study | Normal reactions during hospitalization include anxiety reaction, mild depression, fear of deformity, and a steadily decreasing pain threshold. Potentially pathological in 20-30% of patients include severe depression, severe regression, and delirium. Psychiatric prognosis for children is poorer, with ~80% having complications including fearfulness, suspiciousness, poor school performance, and difficulty in relating to other children. |
|  | Demiralay et al. (2013), Turkey | nNOS expression in the brain of rats after burn and the effect of the ACE inhibitor captopril | Rats, 24 | 30% | Immunohistochemistry | nNOS expression not different in cerebellum and hypothalamus but increased in frontal cortex, striatum and midbrain. ACE inhibitor treatment decreased nNOS in frontal cortex and increased it in striatum and midbrain, no change in hippocampus |
|  | Lestaevel et al. (2003), France | Effects of a thermal injury on brain and blood nitric oxide (NO) content in the rat | Rats, 17 | 20% | Voltammetric method allowing direct and in situ NO measurements | Decrease in cortical NO concentration 24 hours after burn. Blood NO and nitrites increased after burn while hypothalamic inducible NOS2 mRNA decreased |
|  | Li et al. (2001), China | Comparative observation with MRI and pathology of brain edema at the early stage of severe burn | Dogs, 52 | 50% | MRI and pathological examination (gross appearance, electron microscopy and light microscopy). | Brain edema at 12 h, difficult to distinguish gray matter from white matter at boundary line, which became blurred later. Histological changes of brain edema were observed at 6 h after burn accompanied by swelling of endothelial cells and peri-vescular astrocytes, and vacuolation took place in neurons at 12 h with different degrees of necrosis of capillary endothelium, neurons, and axons |
|  | Li et al. (2001), China | Comparative observation with MRI and pathology of brain edema at the early stage of severe burn | Dogs, 52 | 50% | MRI and pathological examination (gross appearance, electron microscopy and light microscopy). | Brain edema at 12 h, difficult to distinguish gray matter from white matter at boundary line, which became blurred later. Histological changes of brain edema were observed at 6 h after burn accompanied by swelling of endothelial cells and peri-vescular astrocytes, and vacuolation took place in neurons at 12 h with different degrees of necrosis of capillary endothelium, neurons, and axons |
|  | Ozaki-Okayama et al. (2004), Japan | Burn injury enhances brain prostaglandin E2 production through induction of cyclooxygenase-2 and microsomal prostaglandin E synthase in cerebral vascular endothelial cells in rats | Rats, 21 | 25% | Analysis of cerebrospinal fluid, brain and the spinal cord using IHC | PGE2 concentration in the cerebrospinal fluid was significantly elevated which was suppressed by a COX-2 inhibitor. COX-2 and PGE2 proteins in vascular endothelial cells throughout CNS. COX-2 and PGE2 co-expressed in perinuclear region of endothelial cells. |
|  | Quan et al. (2001), Japan | Intranuclear ubiquitin immunoreactivity in the pigmented neurons of the substantia nigra in fire fatalities | Humans, 35 | NA | Forensic autopsy cases of fire fatalities | High nuclear ubiquitin positivity in fire fatalities in brain stem, myocardial infarction, and age-dependent increase in Ub-positive % in lower COHb (< 60%) cases. Intranuclear diffuse ubiquitin staining was not observed in cases of high blood cyanide level. |
|  | Reyes et al. (2006), USA | Early inflammatory response in rat brain after peripheral thermal injury | Rats, 14 | 60-70% | mRNA of TNF-α, IL-1β, and ICAM-1 measured with RT-PCR | Up-regulations (5–50 fold) of TNF-α, IL-1β, and ICAM-1 protein level in serum at 7 h, significant increase (3–15 fold) in mRNA expression of TNF-α, IL-1β and ICAM-1 in brain homogenates at 3 h with increased levels remaining at 7 h after injury |
|  | Q. Wang et al. (2011), Japan | Evaluation of human brain damage in fire fatality by quantification of basic fibroblast growth factor (bFGF), glial fibrillary acidic protein (GFAP) and single-stranded DNA (ssDNA) immunoreactivities | Humans, 49 | >20% | Immunohistochemistry | Hight neuronal ssDNA immunopositivity in parietal lobe. Decrease in number of neurons and increase in glia. Higher glial bFGF immunopositivity, low neuronal ssDNA immunopositivity in cerebral cortex and hippocampus |
|  | Zhang et al. (2013), China | Burn injury induces gelsolin expression and cleavage in the brain of mice | Mice, 72 | 15% | Neuropathology was examined by hematoxylin and eosin staining. Cerebral gelsolin mRNA, distribution and cleavage by QPCR, immunohistochemistry and Western blot. Cysteinyl aspartate-specific protease (caspase)-3positive cells and activity measured. | Burn injury could induce pathological alterations in brain including leukocyte infiltration, necrosis, microabscess and gliosis |
| Inflammatory changes affecting sensory and motor pathways | | | | | | |
|  | de Ceballos et al. (1990), UK | Increased [Met]enkephalin and decreased substance P in spinal cord following thermal injury to one limb | Rats, 27 | Hind limb, mild/ severe | [MET]enkephalin levels in lumbar hemisegment of spinal cord using radioimmunoassay after 24 hours | Levels elevated bilaterally by 70%. At 7 days levels elevated only ipsilaterally. Substance P levels decreased by 20-25% bilaterally at 24 hours but not after |
|  | Patwa et al. (2019), USA | Spinal cord motor neuron plasticity accompanies second-degree burn injury and chronic pain | Mice, 28 | Hind paw | Profiled motor neuron dendritic spines in adult mice treated with romidepsin or control at 2 postburn time points | Pak1-inhibitor treatment reduced injury-induced changes to similar levels observed in animals without burn injury. Effectiveness of Pak1-inhibitor was durable, normalized dendritic spine profiles remained for 4 days despite drug withdrawal |
|  | Svoboda et al. (1988), Czechoslovakia | Increase in extracellular potassium level in rat spinal dorsal horn induced by noxious stimulation and peripheral injury | Rat, 55 | Hind paw | Measuring K+ level in dorsal horn using double-barrel K+-sensitive microelectrodes in rats anesthetized with Nembutal | Long-term increase in [K+] in the lower dorsal horn. The K ÷ increases evoked by acute nociceptive stimuli as well as by injury were blocked by pre-injecting the hind paw with 1% procaine. When procaine was applied 20 min or later after injury, the evoked long-term rise in [K+]e was not affected. |
|  | Varga et al. (2021), Hungary | Spinal Excitatory Dynorphinergic Interneurons Contribute to Burn Injury-Induced Nociception Mediated by Phosphorylated Histone 3 at Serine 10 in Rodents | Rats and mice, 24 | Hind legs | Immunohistochemistry, in situ hybridization with the retrograde labelling of projection neurons | Projection neurons only represented a small percentage (5%) of p-S10H3-positive cells, majority was excitatory SDH interneurons. Majority of p-S10H3-expressing dynorphinergic neurons excitatory, as they lacked Pax-2 and showed Lmx1b-immunopositivity. |
|  | White et al. (2011), UK | Severe burn injury induces a characteristic activation of extracellular signal-regulated kinase 1/2 in spinal dorsal horn neurons | Mice, 56 | Hind paw | Immunohistochemistry | phosphorylated ERK1/2 (pERK1/2) immunopositive neurons in the ipsilateral dorsal horn at 5 mins after injury. Activation of ERK1/2 in spinal dorsal horn neurons in mice which lacked the transient receptor potential type 1 receptor (TRPV1) except that the extent to which ERK1/2 was activated in the ipsilateral dorsal horn at 5 min post-injury was significantly greater in wild-type animals when compared to TRPV1 null animals |
|  | Zhang et al. (2022), China | Spinal microglia-derived TNF promotes the astrocytic JNK/CXCL1 pathway activation in a mouse model of burn pain | Mice, 12 | Hind paw | Behavioural tests, immunohistochemistry, RT-PCR | Persistent allodynia and hyperalgesia. Microglia and astrocytes activated in spatially- and temporally-dependent manner in spinal cord. Intrathecal injection of TNF caused apparent pain hypersensitivity, and activation of astrocytes and the upregulation of CXCL1 via the JNK MAPK signalling pathway, indicating that TNF is the key cytokine in the interaction between microglia and astrocytes at the spinal level |
| Changes in the hypothalamic-pituitary-adrenal axis | | | | | | |
|  | Emanuele et al. (2005), USA | The impact of burn injury and ethanol on the cytokine network of the mouse hypothalamus: Reproductive implications | Mice, 16-24 | 15%, full thickness | Measuring serum testosterone, TNFα, IL-1b, IL-6, and LHRH | Serum testosterone decreased in burns with and without ethanol, hypothalamic concentrations of TNFα, IL-1b, and IL-6 increased. Increase by burn greater than that caused by EtOH and the effect of the combined insult was not additive. |
|  | Fuchs et al. (2007), Germany | Cortisol in severely burned patients: investigations on disturbance of the hypothalamic-pituitary-adrenal axis | Humans, 20 | 20% | 20 corticotropin-releasing hormone tests on day 1 after admission | Four patients developed adrenal insufficiency, higher mortality rate in adrenally insufficient patients |
|  | Moiseev et al. (1971), Russia | State of the hypothalamo-hypophyseal system in experimental burns | Rabbits, 7 | 21-38% | Investigation of neurosecretory processes in supraoptic and paraventricular nuclei | In late stages neurosecretory processes restored or degenerative changes develop in neurosecretory cells |
|  | Palmieri et al. (2006), USA | Hypothalamic–Pituitary–Adrenal Axis Response to Sustained Stress after Major Burn Injury in Children | Humans,25 | >20% | Serum total cortisol, ACTH, dehydroepiandrosterone, vasopressin, Pediatric Risk of Mortality score, serum albumin level, and electrolytes | Cortisol level did not correlate with risk of mortality score, albumin, vasopressin, ACTH, or mortality. Adrenal response to stress is intact after severe burn injury, but the ACTH/adrenal feedback loop is disrupted. |
|  | Stoner and Elson (1971), UK | The effect of injury on monoamine concentrations in the rat hypothalamus | Rats, 3-5 | 20% | Measuring NA and 5-HT concentrations in different parts in the brain | Decreased noradrenaline concentration in hypothalamus which recovered slowly if the injury was not fatal. Increased rate of utilization of noradrenaline after injury. These injuries did not affect the 5-hydroxyt~yptamine concentration in the hypothalamus, but changes were found in the concentration of this monoamine and in that of its metabolite in the brain stem. |
| Metabolic changes in the CNS | | | | | | |
|  | Carter et al. (2012), USA | Previous Burn Injury Predisposes Mice to Lipopolysaccharide-Induced Changes in Glucose Metabolism | Mice, 12 | 25% | LPS injection biodistribution measured by weight and radioactivity | 18FDG accumulation significantly different |
|  | Carter et al. (1996), USA | Decreased Cerebral Glucose Utilization in Rats during the Ebb Phase of Thermal Injury | Rats, 18 | 25% | Glucose utilization using ^18^FDG. Oxygen consumption in vivo, and glucose-6-phosphatase and hexokinase activity in vitro | Thermal injury decreases glucose utilization in rat brain during hypometabolic phase partly due to alterations in hexokinase, glucose-6-phosphatase activities, and reductions in oxygen consumption. |
|  | Chance et al. (1987), USA | Burn-Induced Alterations in Feeding, Energy Expenditure, and Brain Amine Neurotransmitters in Rats | NA | 30% | NA | dopamine metabolism was increased in the corpus striatum, nucleus accumbens, and amygdala. Norepinephrine levels elevated in hypothalamus and nucleus accumbens |
|  | Gamelli et al. (1994), USA | Alterations of glucose transporter mRNA and protein levels in the brain following thermal injury and sepsis in mice | Mice, 16 | 15% | Infection, northern blotting and Western blotting | At 4 hours, GLUT1 increased in animals with burn and animals with burns and infection. At 72 hours, GLUT1 abundance was primary response to burn injury and was not altered by wound infection |
|  | Mårtensson et al. (1992), USA | Mitochondrial glutathione in hypermetabolic rats following burn injury and thyroid hormone administration: evidence of a selective effect on brain glutathione by burn injury | Rats, 12 | 50% | Collecting tissue from burn and starved groups to measure effects of stress hormones on mitochondrial GSH | Total GSH levels in peripheral tissues and brain decreased to 50-66% of control levels in burn and starved groups suggesting higher mitochondrial GSH turnover in burned rats than in semi-starved rats. Cerebral cortex mitochondrial GSH levels unaffected by variations in thyroid hormone status |
|  | Green et al. (2016), USA | Central activation of TRPV1 and TRPA1 by novel endogenous agonists contributes to mechanical and thermal allodynia after burn injury | Rats, 20-40 | Hind paw, full thickness | Analysis of lipid extracts of spinal cord tissue with HPLC-MS | Increase in levels of epoxide and diol metabolites of linoleic acid that was reduced after intrathecal injection of oxidative enzyme inhibitor ketoconazole. Activation of TRPV1 and TRPA1. intrathecal injection of ketoconazole reversed post-burn mechanical and thermal allodynia |
|  | Zhang et al. (2008), USA | Burn-Related Metabolic and Signaling Changes in Rat Brain | Rats, 6 | 50% | Tissue lysates analysed for MAPKs activities, insulin receptor substrate (IRS)-1 expression, and Akt activity by western blot and immunoprecipitation | Stimulation of stress responsive components, SAPK/JNK, p38 MAP kinase and p44/42 MAP kinase, and increased IRS-1 expression and Akt activity. No change, however, on the phosphorylation of Ser307 of IRS-1 in brain tissue. |
| Functional changes affecting the periphery | | | | | | |
|  | Coderre and Melzack (1985), Canada | Increased pain sensitivity following heat injury involves a central mechanism | Rats, 29 | Hind paw | Sciatic and saphenous nerve injury and measuring autotomy (self-mutilation) of the limb | Increased pain sensitivity after total nerve sections, and reduced foot-withdrawal latencies in the paw contralateral to injury |
|  | Deveci et al. (2001), Turkey | Clonus: an unusual delayed neurological complication in electrical burn injury | Humans, 4 | Full thickness | EEG, MRI and CT examination | Clonus started 3 weeks after burn and disappeared over a period of 1 yr in 2 patients, and did not disappear in remaining 2 patients |
|  | Guo et al. (2018), USA | Therapeutic potential of Pak1 inhibition for pain associated with cutaneous burn injury | Mice, 47 | Hind paw | Romidepsin injection, behavioural tests, histology and dendritic spine analysis | Romidepsin decreased dendritic spine dysgenesis, reduced c-fos expression, and rescued pain thresholds. Drug discontinuation resulted in relapse of cellular correlates of pain and in lower pain thresholds |
|  | Holavanahalli et al. (2016) | Long-Term Outcomes in Patients Surviving Large Burns: The Musculoskeletal System | Humans, 98 | ~57% | Medical Problem Checklist, subjects also completed the Burn-Specific Health Scale after 17 years (mean) post injury | Joint pain, joint stiffness, problems walking or running, fatigue, and weak arms and hands are conditions that continue to be reported at an average of 17 years from the time of burn injury. |
|  | Joo et al. (2021)South Korea | Frontal lobe oxyhemoglobin levels in patients with lower extremity burns assessed using a functional near-Infrared spectroscopy device during usual walking: a pilot study | Humans, 36 | Various | fNIRS | Increased cortical activation in prefrontal cortex in patients with lower extremity burns |
|  | Ma et al. (2019), USA | Burn-induced Microglia Activation is Associated with Motor Neuron Degeneration and Muscle Wasting in Mice | Mice, 80 | 35% | IHC, Real-time Quantitative PCR, western blot | Activation of microglia, ventral horn motor neurons apoptosis and down-regulation. Burn injury with immobilization disintegrated the pretzel-shaped synapse and was associated with decreased gastrocnemius, tibialis and soleus muscle mass |
|  | Marquez et al. (1993), Canada | Neuropathy in burn patients | Humans, 19 | 10-75 % | Electrophysiological examination, nerve conduction | Generalized axonal neuropathy of the critical illness type seen rarely in spite of systemic complications |
|  | Miao et al. (2020), USA | PGC-1α haploinsufficiency promotes pain chronification after burn injury | Mice, 30 | Hind paw | Hind paw mechanical withdrawal thresholds and thermal withdrawal latency | PGC-1α mice exhibited initial decrease of withdrawal at days 3 and 5 post-injury. PGC-1α+/+ mice fully recovered their withdrawal to preinjury levels by day 11 to 14, PGC-1α+/− mice failed to recover during the same time frame. PGC-1α+/− mice resolved tissue inflammation in similar fashion to PGC-1α+/+ mice |
|  | Nozaki-Taguchi and Yaksh (2002a), Japan | Pharmacology of Spinal Glutamatergic Receptors in Post–Thermal Injury–evoked Tactile Allodynia and Thermal Hyperalgesia | Rats, 400 | Hind paw | Tactile withdrawal thresholds and thermal paw withdrawal latencies | Pretreatment and posttreatment with AMPA–KA antagonists produced a dose-dependent blockade of secondary tactile allodynia. NMDA antagonists, showed little or no effect. Primary thermal hyperalgesia was blocked only by high-dose AMPA–KA antagonists |
|  | Nozaki-Taguchi and Yaksh (2002b), Japan | Spinal and Peripheral μ Opioids and the Development of Secondary Tactile Allodynia After Thermal Injury | Rats, 20 | Hind paw | Mechanical withdrawal thresholds and thermal withdrawal latency | Secondary tactile allodynia reappeared when systemic naloxone was given before, but not after, injury in intrathecal morphine pretreated rats |
|  | Sorkin et al. (2008), USA | Secondary hyperalgesia in the rat first degree burn model is independent of spinal cyclooxygenase and nitric oxide synthase | Rats, >18 | Hind paw | Mechanical withdrawal thresholds | Pre-treatments didn’t cause anti-allodynia. Ziconotide effective in blocking burn-induced sensitivity. L-type and P-type calcium channel blockers produced intermediate effects. |
|  | Sosanya et al. (2019), USA | Involvement of brain-derived neurotrophic factor (BDNF) in chronic intermittent stress-induced enhanced mechanical allodynia in a rat model of burn pain | Rats, 60 | Hind paw | Nociception behaviour testing, Mechanosensitivity assay, Total RNA and protein isolation, Quantitative RT‑PCR analysis, Simple western protein analysis | Increased expression of BDNF mRNA in prefrontal cortex and elevated TrkB and p-TrkB protein levels in hypothalamus in stressed rats with thermal injury but not in stressed or thermally injured rats alone. Administration of CTX-B reduced stress-induced exacerbated mechanical allodynia in burnt rats. |
|  | Sosanya et al. (2017), USA | Sound-stress-induced altered nociceptive behaviors are associated with increased spinal CRFR2 gene expression in a rat model of burn injury | Rats, 24 | Hind paw | Nociceptive behaviour, spinal CRF receptor mRNA expression, plasma corticosterone and spinal CRF concentrations | Increased defecation and transient mechanical allodynia in sound stressed rats. Spinal CRFR2 mRNA expression was unaffected by stress or thermal injury alone, but their combined effect increased its expression. Sound stress had no effect on plasma corticosterone and spinal CRF protein in postburn rats. |
|  | Tan et al. (2013), USA | Burn injury-induced mechanical allodynia is maintained by Rac1-regulated dendritic spine dysgenesis | Rats, 27 | Hind paw | Behavioural tests, profiled dendritic spine shape, density, and distribution of WDR neurons. | Reduced mechanical pain thresholds ipsilateral to the burn injury. Hyperexcitability of ipsilateral WDR neurons in spinal cord dorsal horn. Dendritic spine dysgenesis on ipsilateral WDR neurons exhibiting evidence of neuropathic pain. Administration of Rac1-inhibitor, attenuated dendritic spine dysgenesis, decreased mechanical allodynia and electrophysiological signs of burn-induced neuropathic pain. |
|  | S. Wang et al. (2011), USA | Nociceptive Behavior Following Hindpaw Burn Injury in Young Rats: Response to Systemic Morphine | Rats, 12-18 | <1% | Mechanical withdrawal thresholds, administration of Dextromethorphan, Morphine Antinociception, Immunohistochemistry, Western Blot | Mechanical allodynia, upregulated expression of the NR1 subunit of the N-methyl-d-aspartate (NMDA) receptor, Akt1, Akt2, and protein kinase C g (PKCg), but downregulated expression of neuronal NOS, inducible NOS, and glycogen synthase kinase-3b. Administration of treatment attenuated mechanical allodynia and thermal hyperalgesia |
|  | Wu, Huang, Cheng, et al. (2015), Taiwan | Third-Degree Hindpaw Burn Injury Induced Apoptosis of Lumbar Spinal Cord Ventral Horn Motor Neurons and Sciatic Nerve and Muscle Atrophy in Rats | Rats, 18 | 1% | Apoptosis in ventral horns of lumbar spinal cords, sciatic nerves, and gastrocnemius muscles examined. Schwann cells in sciatic nerve marked with S100. Gastrocnemius muscles harvested to measure denervation atrophy | The VHMNs apoptosis in the spinal cord was observed after inducing third-degree burns in the hind paw. Increase in S100 and TUNEL double-positive cells in the sciatic nerve and gastrocnemius muscle apoptosis and denervation atrophy area |
|  | Wu, Huang, Lo, et al. (2015), Taiwan | Autologous adipose-derived stem cells attenuate muscular atrophy and protect spinal cord ventral horn motor neurons in an animal model of burn injury | Rats, 24 | 1% | Adipose tissue from groin fat pad expanded in culture and labelled. ASCs transplanted into injured hind paw at 4 weeks. Lumbar spinal cord, sciatic nerve, gastrocnemius muscle and hind paw skin processed for immunofluorescent staining at 4 weeks after transplantation, and gastrocnemius muscle was evaluated through use of hematoxylin and eosin staining. | Caspase-3epositive, caspase-9epositive and TUNEL-positive cells significantly increased in dermatome spinal cord VHMNs. Decrease of Schwann cells in sciatic nerve and increase of denervation atrophy in gastrocnemius muscle. ASCs transplantation significantly attenuated apoptotic death of VHMNs and the area of muscle denervation atrophy in gastrocnemius muscle fibres |
|  | Xiang et al. (2017), China | Alkali Burn Induced Corneal Spontaneous Pain and Activated Neuropathic Pain Matrix in the Central Nervous System in Mice | Mice, 17 | Eye | Mechanical threshold in cauterized area tested using Von Frey hairs. Spontaneous pain behaviour investigated with conditioned place preference. ERK investigated in several representative areas of neuropathic pain matrix | Long-lasting damage to corneal sub-basal nerve fibres, spontaneous pain behaviour. phosphor ERK significantly activated in Vc/C1, but not in Vi/Vc. ERK activated in the insular cortex, ACC, and RVM. Pharmacologically blocking ERK activation in ACC abolished alkali burn induced corneal spontaneous pain. |
| Cognitive changes | | | | | | |
|  | Halm et al. (2006), France | Brain and cognitive impairments from burn injury in rats | Rats, 73 | 20% | DPNV, behavioural tests | Cerebral NO depleted for 12 h. During nine days following burn, cognitive test was worse compared to controls |
|  | Purohit et al. (2014), USA | Cognition in Patients With Burn Injury in the Inpatient Rehabilitation Population | Humans, 5347 | ~27.6% | Functional independence measurement (FIM) | Burn patients had lower cognitive scores, memory was most affected |
|  | Zhang et al. (2020), China | Long-lasting neurobehavioral alterations in burn-injured mice resembling post-traumatic stress disorder in humans | Mice, 61 | 15% | Behavioural tests, key molecules expression, spleens for T cell function analysis, and blood for biochemistry and hormones detection | Hyper-arousal state, improved spatial memory in Morris Water Maze test and heightened context fear memory in context fear conditioning, suggesting re-experiencing behaviour. Neurobehavioral alterations associated with increased expression of brain-derived neurotrophic factor along with a remarkable microgliosis and a moderate astrocytosis |
